# LncRNA PSMB8-AS1 contributes to pancreatic cancer progression via modulating miR-382-3p/STAT1/PD-L1 axis

**DOI:** 10.1186/s13046-020-01687-8

**Published:** 2020-09-05

**Authors:** Hao Zhang, Changhao Zhu, Zhiwei He, Shiyu Chen, Lin Li, Chengyi Sun

**Affiliations:** 1 College of Basic Medicine, Guizhou Medical University, Guiyang, China; 2 College of Clinical Medicine, Guizhou Medical University, Guiyang, China; 3 Department of Hepatic-Biliary-Pancreatic Surgery, the Affiliated Hospital of Guizhou Medical University, No.9, Beijing Road, Guiyang, 550000 Guizhou Province China

**Keywords:** PMSB8-AS1, Pancreatic cancer, PDL1, miR-382–3p, STAT1

## Abstract

**Background:**

Accumulating evidence demonstrates the essential role of long non-coding RNA (lncRNA) in various types of cancers, including pancreatic cancer. However, the functions and regulation mechanism of lncRNA PMSB8-AS1 in pancreatic cancer are largely unclear.

**Methods:**

Quantitative reverse transcription PCR (qRT-PCR) is used to examine the expression of PMSB8-AS1 in PC tissues and PC cell lines. The effect of PMSB8-AS1 on the proliferation of PC cells was detected using CCK8 assay, colony assay, and flow cytometry. The effect of PMSB8-AS1 on the migration and invasion of pancreatic cancer cells was detected using a wound-healing assay and transwell migration assay. Bioinformatic analysis, double luciferase reporting assay, western blot, and rescue experiments were used to detect the regulatory relationship between PMSB8-AS1, miR-382–3p, STAT1, and PD-L1.

**Results:**

PMSB8-AS1 expression was upregulated in PC tissues and cell lines and positively associated with the worst survival in patients with PC. The in vitro and in vivo assays demonstrated that overexpression of PMSB8-AS1 significantly promoted pancreatic cancer cell proliferation, migration, and invasion, whereas knockdown of PMSB8-AS1 suppressed cell proliferation, migration, invasion, and EMT, and decreased apoptosis of PC cells. Besides, PMSB8-AS1 directly bound to miR-382–3p downregulated its expression. Besides, PMSB8-AS1 reversed the effect of miR-382–3p on the growth and metastasis of PC cells, which might be targeted on STAT1. Furthermore, STAT1 is the transcriptional factor that activates the expression of PD-L1.

**Conclusion:**

lncRNA PMSB8-AS1 promotes pancreatic cancer progression via STAT1 by sponging miR-382–3p involving regulation PD-L1.

## Background

Pancreatic cancer (PC) is one of the major causes of cancer-related deaths worldwide [1, 2]. Although there are many therapeutic approaches, such as chemotherapy and radiofrequency, surgical resection is the only effective treatment. However, the early diagnosis of PC is a key factor for surgery. Hence, understanding the biological processes and molecular mechanisms underlying PC progression might contribute to the effective treatment of PC.

Long non-coding RNAs (lncRNAs) exert an essential role in the occurrence and progression of tumors [3, 4], mainly in cancer proliferation, metastasis, and other biological functions associated with tumorigenesis [5, 6]. In PC, abnormal expression and dysfunction of lncRNAs are shown to be associated with rapid progression of cancer and distant metastasis [7, 8]. A large number of studies have shown that lncRNA can participate in the regulation of various biological functions of tumors such as epigenetics, post-transcriptional regulation, genomic stability, and ceRNA regulation mechanism [9–11]. For example, Wang et al. [12] reported that UCA1 increased PDL1 expression from the repression of miR-26a/b, miR-193a, and miR-214, contributed to the gastric cancer cell proliferation, migration, and immune escape. Besides, lncRNA NONHSAT101069 was overexpressed in breast cancer tissues and promoted epirubicin resistance, migration, and invasion of breast cancer cells through regulation of NONHSAT101069/miR-129–5p/Twist1 axis in breast cancer [13]. Moreover, the role of lncRNA in epigenetics was usually reported in various cancers. A previous study showed that lncRNA GLS-AS mediates a feedback loop between Myc and GLS, providing a potential therapeutic target for metabolic reprogramming in pancreatic cancer. PMSB8-AS1, researched in this study, could be a new host factor target for developing antiviral therapy against influenza virus [14]. The bioinformatic analysis provides new, potential prognostic biomarkers, and therapeutic targets for PDAC that need to be further investigated [15]. However, the function and molecular mechanism of lncRNA PSMB8-AS1 remain unclear.

It is well known that the competing endogenous RNA (ceRNA) regulation network is the most frequently reported molecular mechanism to elucidate the function in tumor biology progress [16–19]. Therefore, miRNA as the pivotal downstream of lncRNA mediated tumorigenesis via regulation of the target gene. For example, lncRNA GAS5 upregulated the expression of PTEN by functioning as a ceRNA of miR-222–3p, regulating the PC cell migration, invasion, and autophagy [20]. Based on current research findings, we speculated PSMB8-AS1 probably acted as a sponge regulating miRNAs exerted corresponding biological function.

In this study, we found that PSMB8-AS1 and STAT1 are existing binding sites of miR-382–3p, and demonstrated that PSMB8-AS1 promotes the expression of STAT1/PD-L1, forming a new theoretical basis for pancreatic cancer progression and providing a possible approach for targeted therapy.

## Methods

### Patients and clinical samples

Ninety paired pancreatic cancer and adjacent non-cancer specimens were collected from patients in our department. The patients had undergone surgery at the Hepatic-Biliary-Pancreatic surgery department of Guizhou Medical University affiliated Hospitals between 2015 and 2018. We have received approval from the Institutional Review Board of Guizhou Medical University affiliated Hospital before sample collection. The collected tissues were quickly stored in a − 80 °C freezer for the detection of expression. The clinicopathological characteristics of patients are shown in Supplementary Table 1.

### Cell culture and chemicals

Pancreatic cancer cell lines were obtained from the American Type Culture Collection. The pancreatic ductal epithelial cells were obtained from the China Center for Type Culture Collection of Wuhan University. The cell culture medium consisted of RPMI 1640 Medium (Gibco, USA) and DMEM Medium supplemented with 10% fetal bovine serum (FBS) (Gibco, USA), 100 μg/mL streptomycin, and 100 IU/mL penicillin. All cell lines were maintained at 37 °C in a humidified incubator with 5% CO_2_.

### RNA extraction and PCR assays

The total RNA extraction of tissues and cells was performed using RNA extraction kits (QIAGEN, Germany) according to the manufacturer’s protocol. RT-qPCR was performed using the Bio-Rad CFX96 Real-Time PCR operating instrument with SYBR qPCR Mix (Takara, Dalian, China). The lncRNA and mRNA were normalized to the levels of GAPDH, and the miRNA was normalized to the levels of U6. The primers used for real-time PCR are shown in Supplementary Table 2.

### Cell transfection

Target specific shRNA of PSMB8-AS1, STAT1, PD-L1, and the miRNA mimic, inhibitor, were synthesized and purchased from Ribobio (Guangzhou, China). All products were compared to the corresponding negative control. Transfection of this well-established shRNA mimicking into pancreatic cancer cells was performed using LipofectAMINE 2000 reagent (Invitrogen, USA). The corresponding shRNA, mimic, inhibitor sequences are shown in Supplementary Table 2.

### RNA–fish

The indicated pancreatic cancer cells were seeded in the confocal culture plate. Cy3-labeled PSMB8-AS1 probes were synthesized and purchased from Ribobio (Guangzhou, China). The cell was fixed with 4% paraformaldehyde, and the fluorescent staining was done according to the manufacturer’s instructions. The images were recorded and analyzed using the CAIS Laser Scanning Confocal Microscope (Nikon Instruments Inc., Japan).

### Luciferase reporter assay

PC cells were plated into the 6-well plates at the dentist of 5 × 10^5^. Then the miR-382–3p mimic was transfected into the pancreatic cancer cell. After removing the supernatant, the lysate was used to measure the firefly and Renilla luciferase activities. According to the manufacturer’s protocol (Ribobio, Guangzhou), the corresponding absorbance was detected using the microplate reader. Then, the relative luciferase activity was normalized to the firefly luciferase internal control.

### Colony formation assay

The indicated cells were digested and resuspended and counted under a microscope. And the cells were cultured in 6 cm plates at a density of 1500 cells per well. The cells were cultured under normal culture conditions for 14 days. The supernatant was removed, and the cells were fixed with paraformaldehyde, the cells were dyed with the purple crystal. Then, the plates were washed with PBS thrice and photographed.

### Proliferation and metastasis in vivo

A 4-week-old female BALB/c nude mice and PANC-1 cells were used for tumor xenografts experiments. First, overexpressed PSMB8-AS1 and control lentivirus were transfected into PANC-1 cells. Then, the indicated PANC-1 cells were expanding cultivated and subcutaneously injected into the left side flank of the nude mice (5 × 10^6^, 200 μL). The volume of tumor xenografts was measured every week. The mice were euthanized, and images were recorded. IHC was used to detect the expression of KI67 and PCNA of the tumor xenografts tissue. For the metastasis assays, first, the indicated PANC-1 cells were expanding cultivated and subcutaneously injected into the right spleen of nude mice (5 × 10^6^, 200 μL). IHC was used to detect the expression of KI67 and PCNA of the tumor xenografts tissue and H & E staining for the mice liver tissue to evaluate the proliferation and metastasis area. The animal experiments were approved by the Animal Care Committee of Guizhou University.

### Western blotting analysis

The indicated cells were washed with pre-cool PBS thrice; the plate was added into RIPA lysis buffer (Boster, Wuhan, China) for 30 min. Then the protein lysate was centrifuged at 12,000 rpm, and the protein concentration was determined using the BCA protein assay kit (Beyotime). The lysis and the 5× loading buffer were mixed and boiled at 95 °C for 5 min. The total protein was separated by electrophoresis and transferred onto PVDF membranes. The membranes were incubated with the corresponding primary antibodies for 16 h. The membranes were blocked for 1 h in the 5% TBST milk, then incubated with the specific HRP-conjugated secondary antibodies at room temperature for 2 h. All membranes were captured by using the Bio-Rad Imaging System.

## Results

### PMSB8-AS1 expression increases in PC tissue and cell lines

**Fig. 1:**
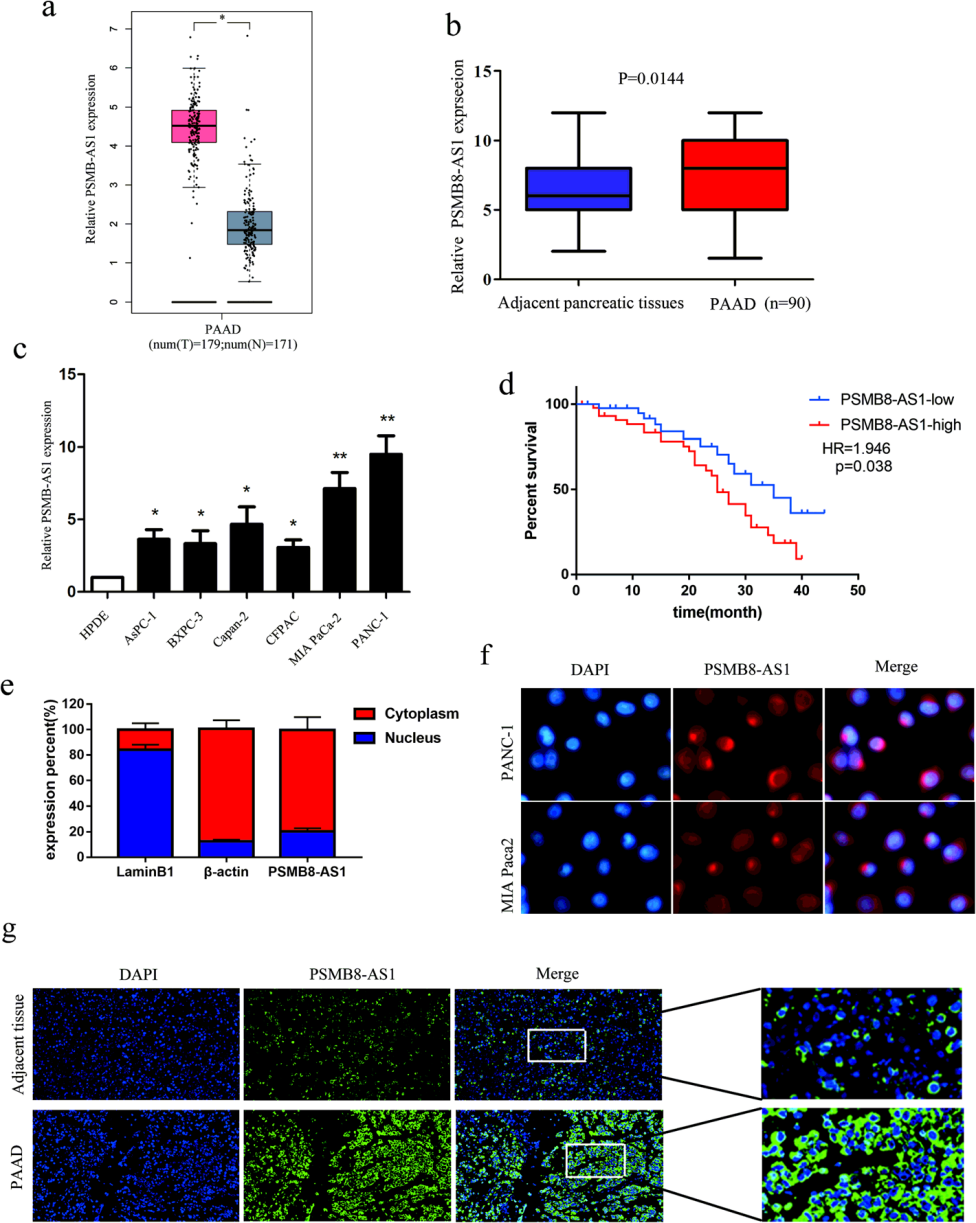
PMSB8-AS1 expression increases in PC tissue and cell lines. **a** Bioinformatic analysis of the expression of PMSB8-AS1 using TCGA database. **b** qRT-PCR to determine the expression of PMSB8-AS1 in 90 paired PC tissues and matched normal tissues. **c** qRT-PCR to determine the expression of PMSB8-AS1 in six of the nine PC cell lines compared in the HPDE normal pancreatic epithelial cell line. **d** Kaplan-Meier curve showing survival in PC patients divided by PSMB8-AS1 expression. **e** qRT-PCR analysis of RNA obtained from cell nuclei and cytoplasm of PC cells. **f-g** FISH assay analysis for the location of PMSB8-AS1 in pancreatic cancer cell and tissues

**Fig. 2:**
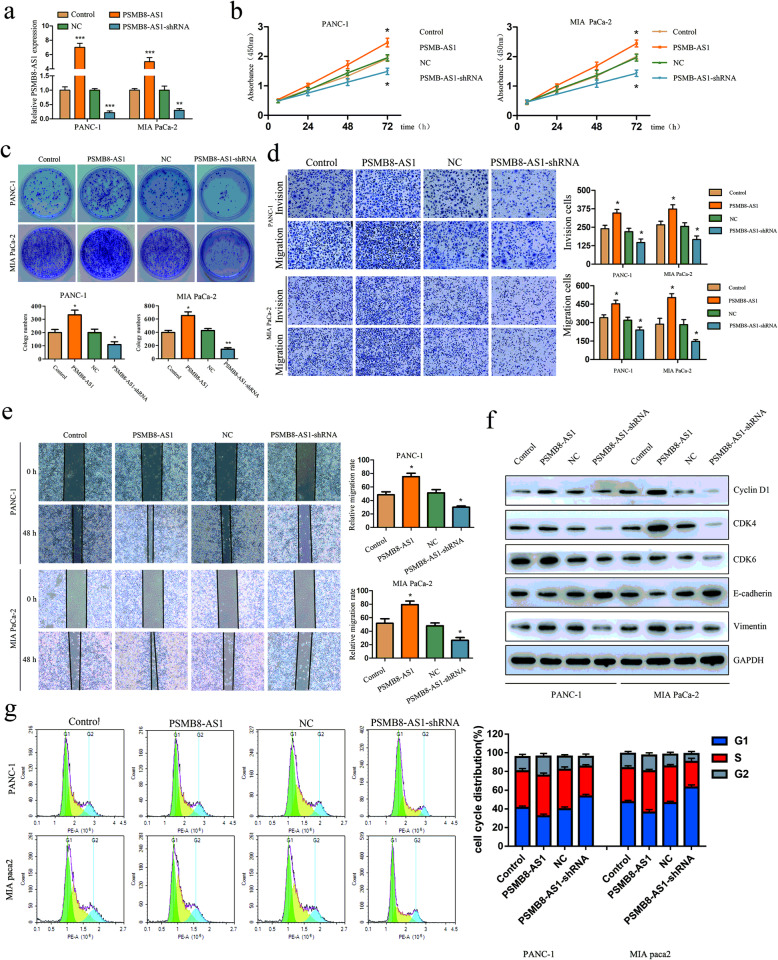
PMSB8-AS1 promotes PC cell proliferation and metastasis in vitro. **a** The qRT-PCR was performed to confirm the transfection efficiency of PSMB8-AS1 overexpressed and underexpressed lentivirus plasmid in PANC-1 and MIA-paca2 cells. **b** CCK-8 assay of PSMB8-AS1 knockdown and control group PC cells at the indicated times. **c** Plate clone formation assays were performed to evaluate cell invasion in PSMB8-AS1 overexpressed and knockdown PC cells. **d** Cell scratch assay for the migration ability of the indicated PC cells. **e** Transwell experiments were performed to analyze the cell migration and invasion in PSMB8-AS1 overexpressed and knockdown PC cells. **f** Western blot analysis to determine expression level of the EMT and cell cycle relative marker in PSMB8-AS1 overexpressed and knockdown PC cells. **g** Effects of PSMB8-AS1 overexpression and knockdown on cell cycle progression in PC cells

To explore the potential involvement of lncRNAs in pancreatic cancer, we analyzed the lncRNA expression analysis across the TCGA database. Our results showed that PMSB8-AS1 was significantly highly expressed in pancreatic cancer samples than in normal samples, as a potential target for future treatment (Fig. 1a). We used qRT-PCR to determine the expression of PMSB8-AS1 in 90 paired PC tissues and matched with normal tissues. As shown in Fig. 1b, the expression of PMSB8-AS1 in PC tissues was markedly increased than in normal tissues (*p =* 0.0144). Meanwhile, PMSB8-AS1 expression was significantly higher in six of the nine PC cell lines than in the HPDE normal pancreatic epithelial cell line (Fig. 1c). Further, Kaplan–Meier analysis illustrated that the PSMB8-AS1 expression highly patients had poorer prognosis than those PSMB8-AS1 expression lowly patients (*P* = 0.0038, HR = 1.946 (Fig. 2d). The nuclear extracted RNA fractionation demonstrated that PSMB8-AS1 was mainly located in the cytoplasm (Fig. 1e). The FISH assay was used to analyze the location of PSMB8-AS1 in the pancreatic cancer cell and tissues, and the result indicated that PSMB8-AS1 mostly located in the cytoplasm (Fig. 1d). In the different clinical subgroups, PMSB8-AS1 expression was induced in patients with tumor size (T stage), lymphatic metastasis, and advanced TNM stage (stage III-IV) (Table 1). Taken together, these data suggest that the induced expression of PMSB8-AS1 is commonly observed in PC and may be involved in PC progression and metastasis.

### PMSB8-AS1 promotes PC cell proliferation in vitro

Next, we explored the functional effects of PSMB8-AS1 on PC cells. The PC cells were transfected with PSMB8-AS1-shRNA and functional PSMB8-AS1-cDNA. The qRT-PCR results confirmed that the expression of PSMB8-AS1 was effectively modulated in PANC-1 and MIA-paca2 cells (Fig. 2a). The CCK8 and plate clone formation assay were used to examine the proliferation of PC cells. The CCK-8 and plate clone formation indicated that PSMB8-AS1 overexpressed PC cell leading to a significant increase in proliferation; however, PSMB8-AS1 knockdown showed the opposite result in downregulating cells (Fig. 2b, c). We then examined the invasion and migration ability of stable transfected cells using cell scratch and transwell assay. The transwell assay suggested that PSMB8-AS1 overexpression induced cell migration and invasion, whereas knockdown of PSMB8-AS1 significantly inhibited cell migration and invasion in PANC-1 and MIA-paca2 cells (Fig. 2d, e). To investigate the underlying regulator of PSMB8-AS1 that induces cell proliferation and metastasis, the cell cycle and EMT associated protein were detected using western blot assay, including E-cadherin, Vimentin, cyclin D1, CDK6, and CDK4. The results demonstrated that Vimentin, cyclin D1, CDK6, CDK4 were upregulated, and E-cadherin was downregulated in PSMB8-AS1 overexpression PC cells, the expression of these indicators was reversed in downregulating cells (Fig. 2f). The cell cycle assay indicated that PMSB8-AS1 overexpressed shown significantly increase in PC cells in G0/G1 phase. While, PMSB8-AS1 knockdown resulted in a significantly cycle block in PC cells in G0/G1 phase (Fig. 2g). Taken together, these results indicated that PSMB8-AS1 exerted an oncogene through promoted proliferation and metastasis in PC cells.

### PMSB8-AS1 promotes growth and metastasis of pancreatic cancer in vivo

**Fig. 3:**
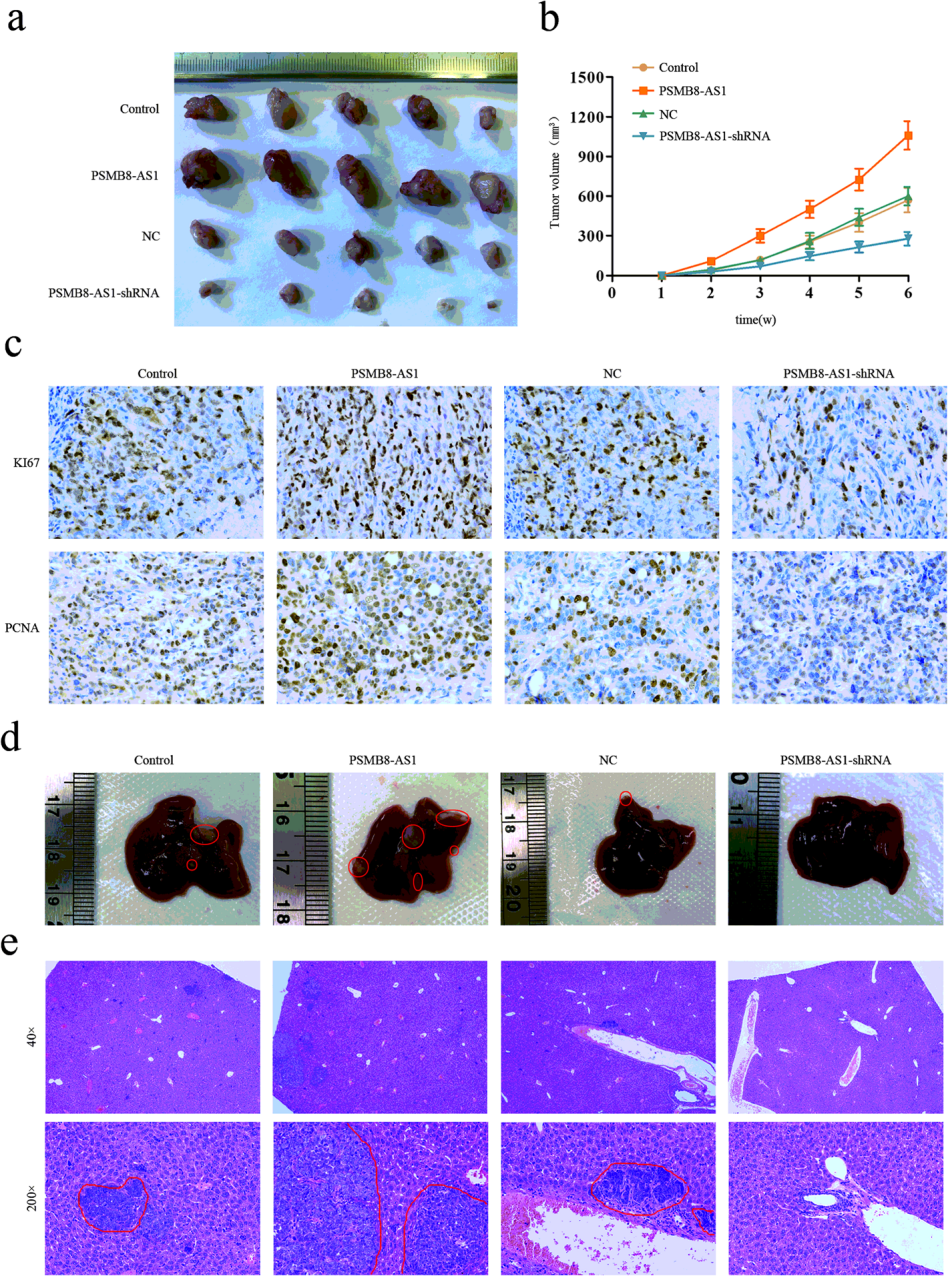
PMSB8-AS1 promotes growth and metastasis of pancreatic cancer in vivo. **a** Nude mice were given xenografts of PSMB8-AS1 overexpressed and knockdown PANC-1 cells (5 × 10^6^ cells per site). The tumors were dissected and photographed after approximately six weeks (*n* = 5 per group). **b** The growth curve of PSMB8-AS1 overexpressed and knockdown tumors compared to the control group. **c** The IHC was performed to analyze the KI-67 and PCNA in the PSMB8-AS1 overexpressed and knockdown xenograft tissues. **d** Representative images of the liver of PSMB8-AS1 with overexpressed and knockdown tumors compared to the control group. **e** Representative images of HE staining in metastatic nodules in the lungs of nude mice. The metastatic nodules are indicated by red line

To determine whether PMSB8-AS1 was involved in tumor proliferation, metastasis in vivo, PMSB8-AS1 overexpression, knockdown PANC-1 cells, and control group were performed to establish a xenograft tumor formation model and a spleen xenograft tumor metastasis model. The indicated groups of PANC-1 cells were injected subcutaneously into the left side flanks of five nude mice. The tumor volume increased rapidly, and the tumor weight was higher at the 6th week in the PSMB8-AS1 overexpressed group compared with the control group, whereas the PSMB8-AS1 knockdown group showed the inverse results (Fig. 3a). The results indicated that PSMB8-AS1 overexpression remarkably promoted the tumorigenic ability, while PSMB8-AS1 knockdown inhibited the tumorigenic ability of PC cells in vivo (Fig. 3b). The IHC assay showed that proliferation markers, KI-67 and PCNA, were overexpressed in the PSMB8-AS1 overexpressed group xenograft, and in the PSMB8-AS1 knockdown group exerted lower expression (Fig. 3c). The liver metastasis model indicated that the PSMB8-AS1 overexpression group exerted more metastasis regions, less in the PSMB8-AS1 knockdown group (Fig. 3d, e).

### PSMB8-AS1 activity is directly and partially negatively regulated by miR-382–3p

**Fig. 4:**
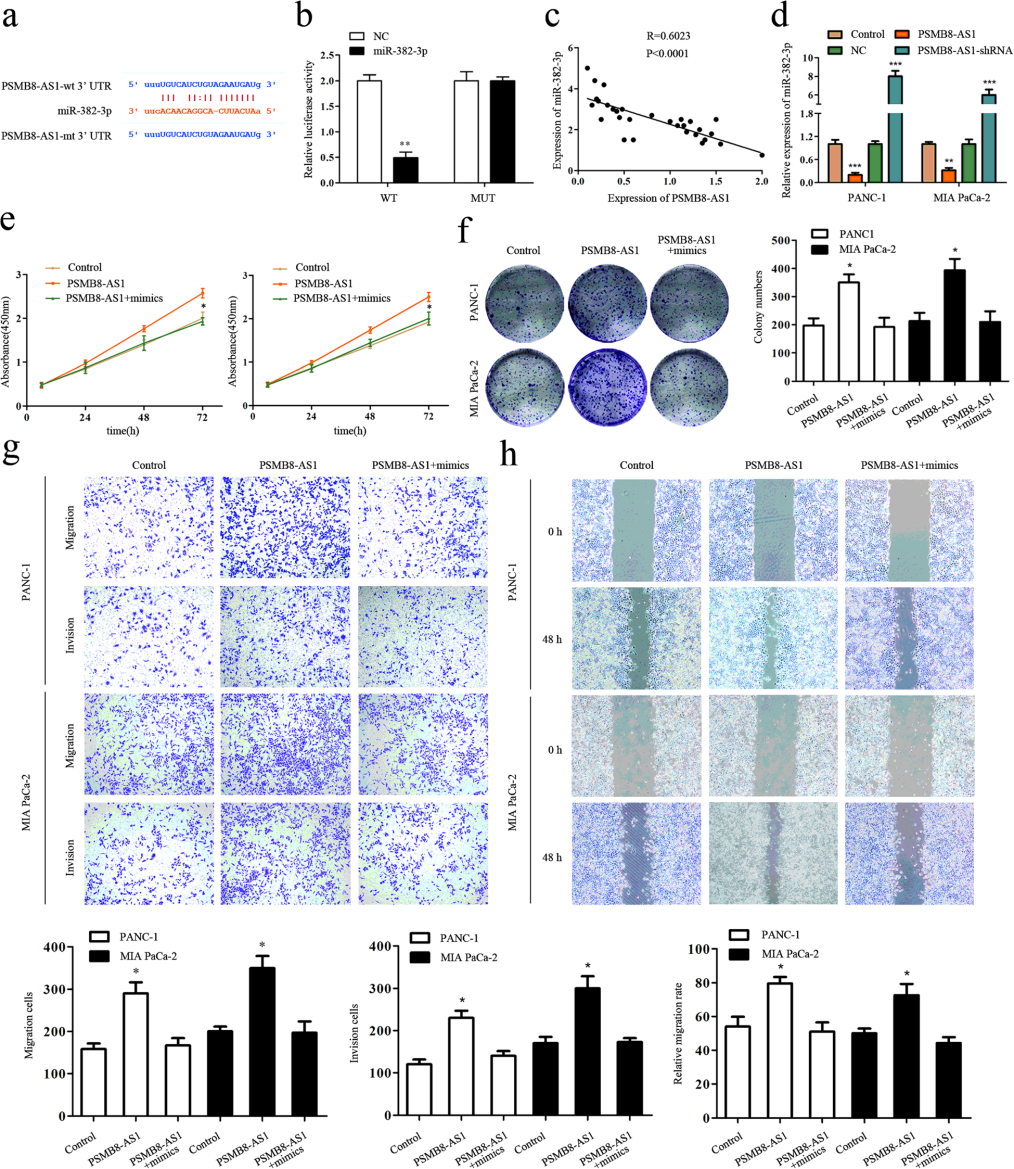
PSMB8-AS1 activity is directly and partially negatively regulated by miR-382-3p. **a** Bioinformatic prediction of the potential binding site of miR-382-3p and PSMB8-AS1. **b** Luciferase reporter assay analysis for the interaction with miR-382-3p and PSMB8-AS1. **c** Spearman’s rank correlation showing the negative correlation with miR-382-3p and PSMB8-AS1. **d-e** RT-qPCR assay indicating that PSMB8-AS1 was downregulated in the miR-382-3p overexpression PANC-1 and Mia paca2 cells. **e-f** CCK8 and clone overexpressing miR-382–3p partly inhibited the proliferation promoted by PSMB8-AS1. **g-h** Cell scratch and transwell assay showing that overexpressing miR-382–3p partly restrained the proliferation promoted by PSMB8-AS1

Accumulated research reported that lncRNAs might sponge miRNA to regulate gene expression. Therefore, we explore the potential miRNAs associated with PSMB8-AS1. Bioinformatic tools such as Starbase (starbase.sysu.edu.cn) was used to predict the potential functional target miRNAs that could bind with the PSMB8-AS1 sequence (Fig. 4a). Then, we constructed the plasmids of wild-type (PSMB8-AS1-WT) and mutated (PSMB8-AS1-Mut) miR-382–3p binding site into dual-luciferase reporters. After transfected with wild-type or mutated dual-luciferase reporters, we detected the luciferase activity in the control and miR-382–3p. The luciferase activity assay showed that the relative luciferase activity of PSMB8-AS1-WT in PANC-1 and Mia-paca2 cells was inhibited after the co-transfection of miR-382–3p mimic but did not change the activity of mutant plasmid, which suggested that miR-382–3p is a direct target of PSMB8-AS1 (Fig. 4b). The qPCR assay indicated that PSMB8-AS1 and miR-382–3p were negatively correlated (Fig. 4c). Furthermore, qPCR assay indicated that miR-382–3p was downregulated in the PSMB8-AS1 overexpression PANC-1 and Mia-paca2 cells (Fig. 4d). To determine whether miR-382–3p played a role in lncRNA PSMB8-AS1 mediated oncogenic role of PC cells, PANC-1 and Mia-paca2 cells were co-transfected with lncRNA PSMB8-AS1 shRNA and miR-382–3p inhibitor. Results demonstrated that the increase in cell proliferation and invasion mediated by lncRNA PSMB8-AS1 upregulation could be partially rescued by miR-382–3p mimic (Fig. 4e–h). These data showed that lncRNA PSMB8-AS1 promotes the tumor progress phenotype in part, by regulating miR-382–3p.

### STAT1 is a functional target of miR-382–3p and regulated by PSMB8-AS1

**Fig. 5:**
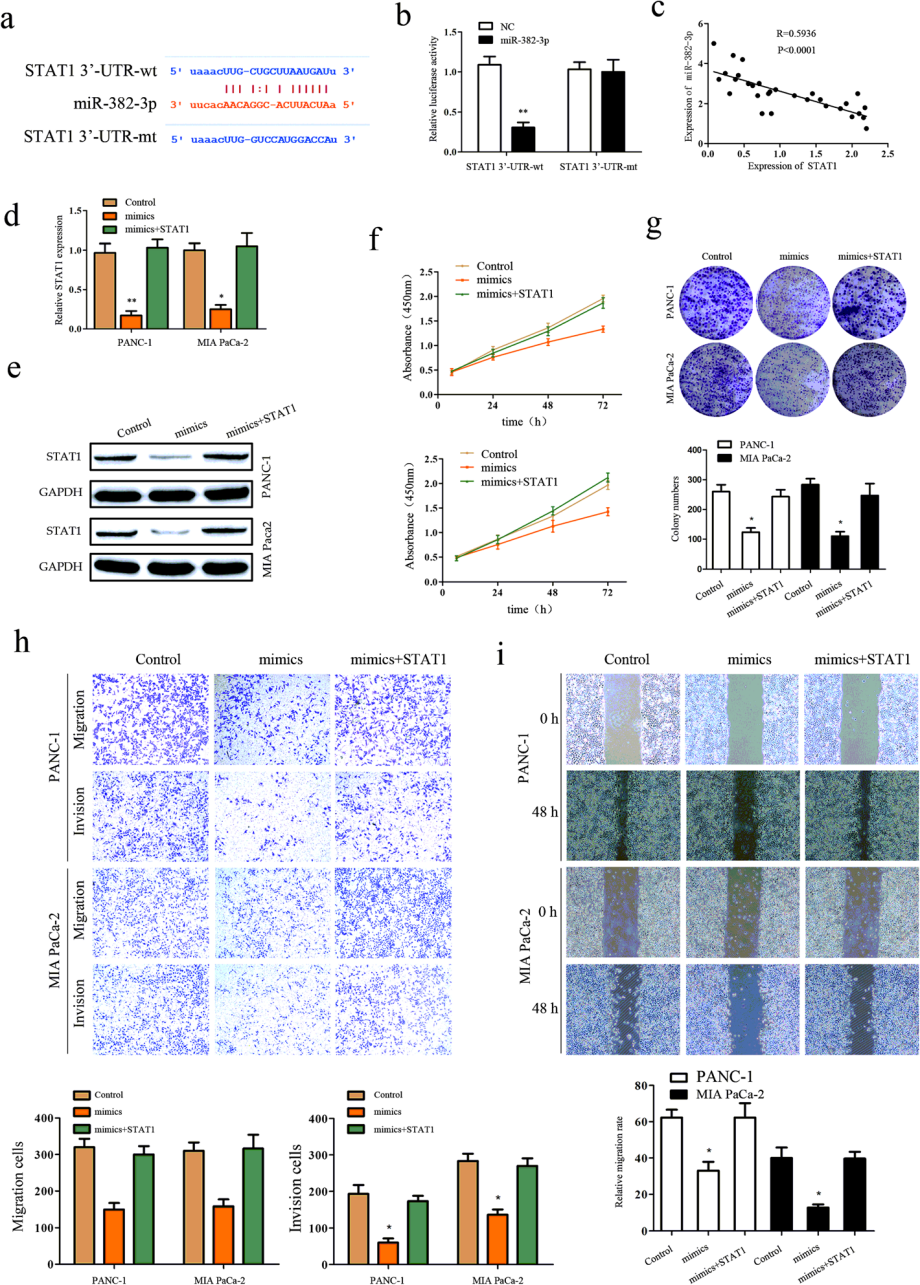
STAT1 is a functional target of miR-382–3p and regulated by PSMB8-AS1. **a** Bioinformatic prediction of the potential binding site of miR-382–3p and STAT1. **b** Luciferase reporter assay analysis showing that the interaction with miR-382–3p and STAT1. **c** Spearman’s rank correlation showing the negative correlation between miR-382–3p and STAT1. **d-e** qPCR and western blot assay indicating that STAT1 was downregulated in the miR-382–3p overexpression PANC-1 and Mia paca2 cells. **f-g** CCK8 and clone formation eluted miR-382–3p overexpress could partly restrained the proliferation promoted by STAT1. **h-i** Cell scratch and transwell assay eluted miR-382–3p overexpress could partly restrained the migration and invasion promoted by STAT1

To select the functional target of PSMB8-AS1, miR-382–3p, we used online bioinformatic tools such as Starbase (starbase.sysu.edu.cn) to analyze and select STAT1 as the downstream target gene of miR-382–3p (Fig. 5a). Next, the luciferase activity assay was used to verify whether STAT1 mRNA 3′-UTR could combine with the miR-382–3p. The STAT1-wt-3′-UTR, STAT1-mt-3′-UTR, miR-382–3p mimic were transfected into the PANC-1, and Mia-paca2 cells, the luciferase activity of STAT1-wt-3′-UTR was significantly reduced by miR-382–3p mimic compared with negative control, but this suppression effect was not observed in the STAT1-mt-3′-UTR (Fig. 5b). Also, an inverse association was found between the expression of miR-382–3p and STAT1 (Fig. 5c). Overexpression of miR-382–3p significantly decreased the mRNA and protein express level of STAT1 in PANC-1 and Mia-paca2 cells, as overexpression of STAT1 could reduce the inhibition in PANC-1 and Mia-paca2 cells (Fig. 5d-e). We determined whether STAT1 was a functional target of miR-382–3p in PANC-1 and Mia-paca2 cells. The growth and metastasis assay demonstrated that overexpression of STAT1 could significantly reverse the effect of miR-382–3p in PC growth and metastasis. Results demonstrated that the promotion of cell growth and metastasis mediated by miR-382–3p downregulation could be partially rescued by STAT1 overexpression (Fig. 5f–i). These results suggest that miR-382–3p regulates STAT1 expression through post-transcriptional modulation to regulate PC progress.

### PSMB8-AS1 promotes pancreatic cancer progression through regulating STAT1

**Fig. 6:**
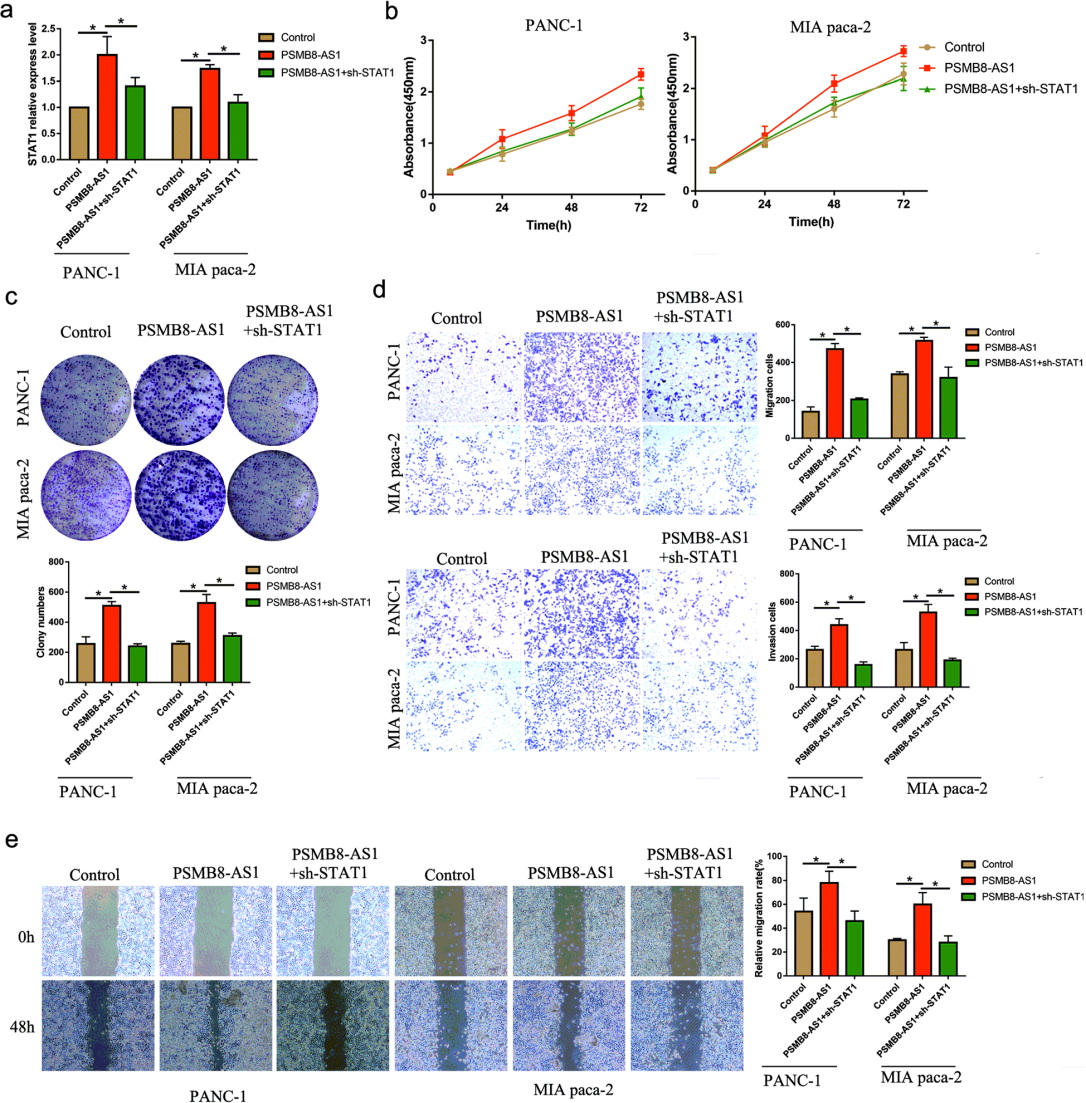
PSMB8-AS1 promotes pancreatic cancer progression through regulating STAT1. **a** The q-PCR of the expression of STAT1 affected by PSMB8-AS1 and STAT1 shRNA. **b-c** CCK8 and plate clone assays were performed to examine the growth in PSMB8-AS1 overexpressed and STAT1 downregulation PC cells. **d-e** Transwell and cell scratch assays were performed to examine the migration and invasion of PSMB8-AS1 overexpressed and STAT1 downregulation PC cells

As STAT1 was as a transcriptional factor of PD-L1, we further explored the PSMB8-AS1-dependent regulation of STAT1 on PD-L1 expression. Based on the TCGA database, we found that both PSMB8 and STAT1 were positively correlated (Supplementary Fig. S1a). The q-PCR assay analyzed the expression of STAT1 affected by PSMB8-AS1 and STAT1 shRNA; the results showed that STAT1 was upregulated in the PSMB8-AS1 overexpressed group, and was reversed by STAT1 shRNA (Fig. 6a).

Next, we explored whether the STAT1 expression affects the proliferation and metastasis in PSMB8-AS1 overexpressed PC cells. CCK8 and plate clones assays were used to examine the growth of PSMB8-AS1 overexpressed PC cells and co-transfected with sh-STAT1, the results demonstrated that the growth was increased in the PSMB8-AS1 group, and was partially reversed in the PSMB8-AS1 overexpressed and co-transfected with sh-STAT1 (Fig. 6b,c). Next, we found that PSMB8-AS1 significantly promotes the migration and invasion ability, and the downregulated STAT1 partly restrained the effect of PSMB8-AS1 overexpression (Fig. 6d-e).

### STAT1 transcriptionally regulated PD-L1 to inactivate CD8+ T cells

**Fig. 7:**
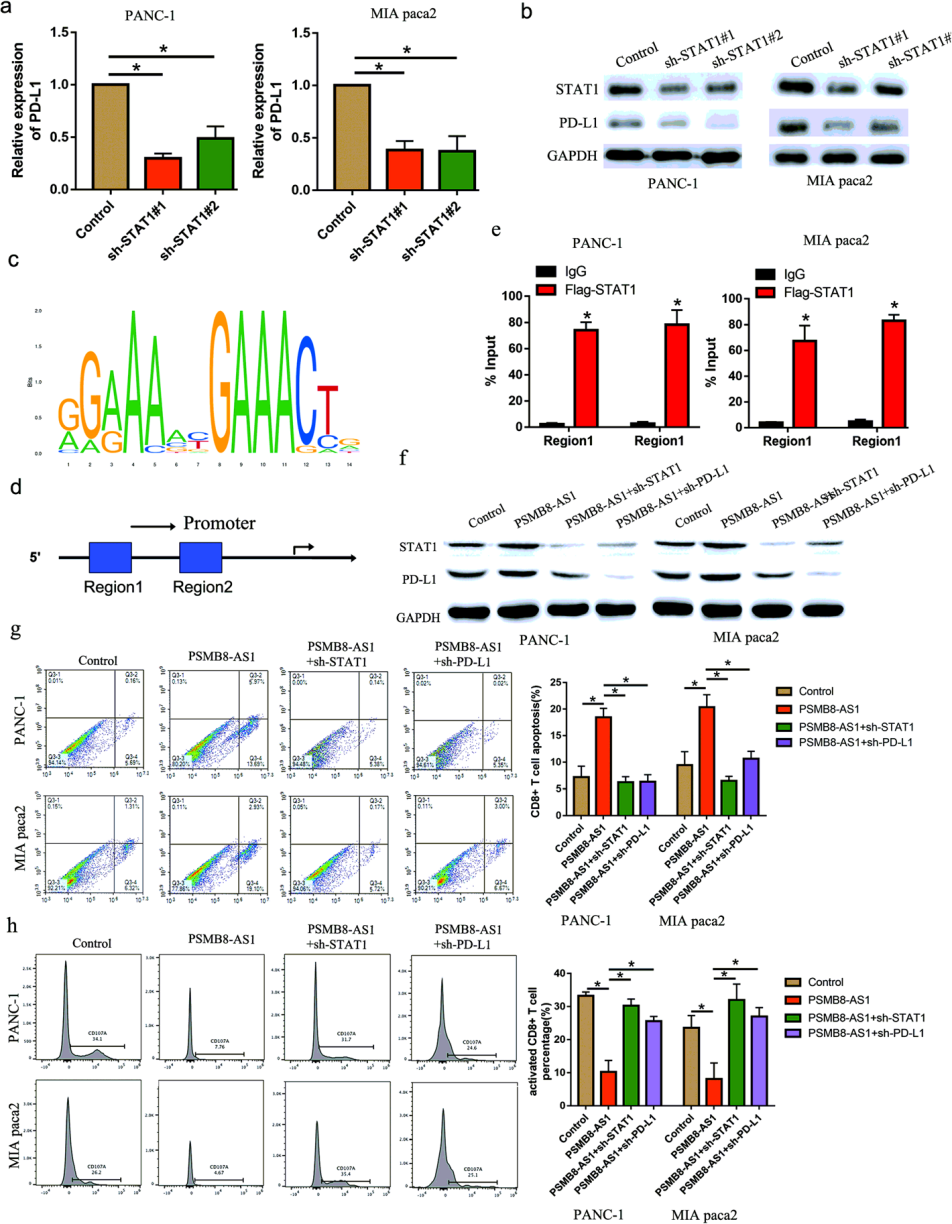
STAT1 transcriptionally regulated PD-L1 to inactivate CD8+ T cells. **a-b** qPCR and western blot assay indicating that PD-L1 was downregulated in the STAT1 knockdown PANC-1 and Mia paca2 cells. **c-d** Bioinformation analysis of the promoter binding sites of STAT1. **e** CHIP assay was performed to analysis the binding of STAT1 and PD-L1 promoter. **f** Western blot analysis for the expression level of the PD-L1 and STAT1 in PSMB8-AS1 overexpressed, STAT1 and PD-L1 downregulation PC cells. **g** Flow cytometric analysis of the apoptosis of CD8+ T cells in PSMB8-AS1 overexpressed and, STAT1 and PD-L1 downregulation PC cells. **h** Flow cytometric analysis of the activity of CD8+ T cells in PSMB8-AS1 overexpressed and, STAT1 and PD-L1 downregulation PC cells

Previous literature demonstrated that the tumor microenvironment provided the space for tumor cells and immune cells to interact with each other. Next, we analyzed the correlation between STAT1 and PD-L1 expression in the TCGA database, we found that STAT1 was positively correlated with PD-L1 in the pancreatic cancer tissue (Supplementary Fig. S1b). The RT-qPCR and western-blot assay were performed to verified whether STAT1 knockdown inhibit the PD-L1 expression level, the results indicated PD-L1 expression was markedly inhibited in the STAT1 knockdown PC cell in mRNA and protein level (Fig. 7a-b). According to the prediction website (http://jaspar.genereg.net/), the transcriptional bind site was GGAAA and GAACT (Fig. 7c). Then, we examined whether the binding site located in PD-L1 promoter regions. Interestingly, the PD-L1 promoter contains two putative STAT1 DNA-binding motifs located at -309 bp (Region 1) and − 1210 bp (Region 2) relative to the transcription start site (Fig. 7d). Further, the CHIP experiment was performed to illustrated that STAT1 could bind in the promoter of PD-L1 in the two motifs (Fig. 7e). The western-blot assay showed that PD-L1 was upregulated in the PSMB8-AS1 overexpressed PC cells, and partly downregulated in overexpressed PSMB8-AS1 co-transfected with STAT1 or PD-L1 inhibition (Fig. 7f). Further, we detected the effect of PSMB8-AS1/STAT1 on the activity and function of CD8 + T cells in PC. Flow cytometric analysis revealed that PSMB8-AS1 significantly promoted the apoptosis of CD8+ T cells and decreased the activaty of CD8+ T cells, and the effect was partly restrained in co-transfected with sh-STAT1 (Fig. 7g, h). These results suggest that PSMB8-AS1-dependent regulation of STAT1 is a key mediator of PD-L1 expression upregulation.

### The positive correlation between PSMB8-AS1 and miR-382-3p/STAT1/PD-L1 axis in tumor tissues from xenografts and patients with PC

**Fig. 8:**
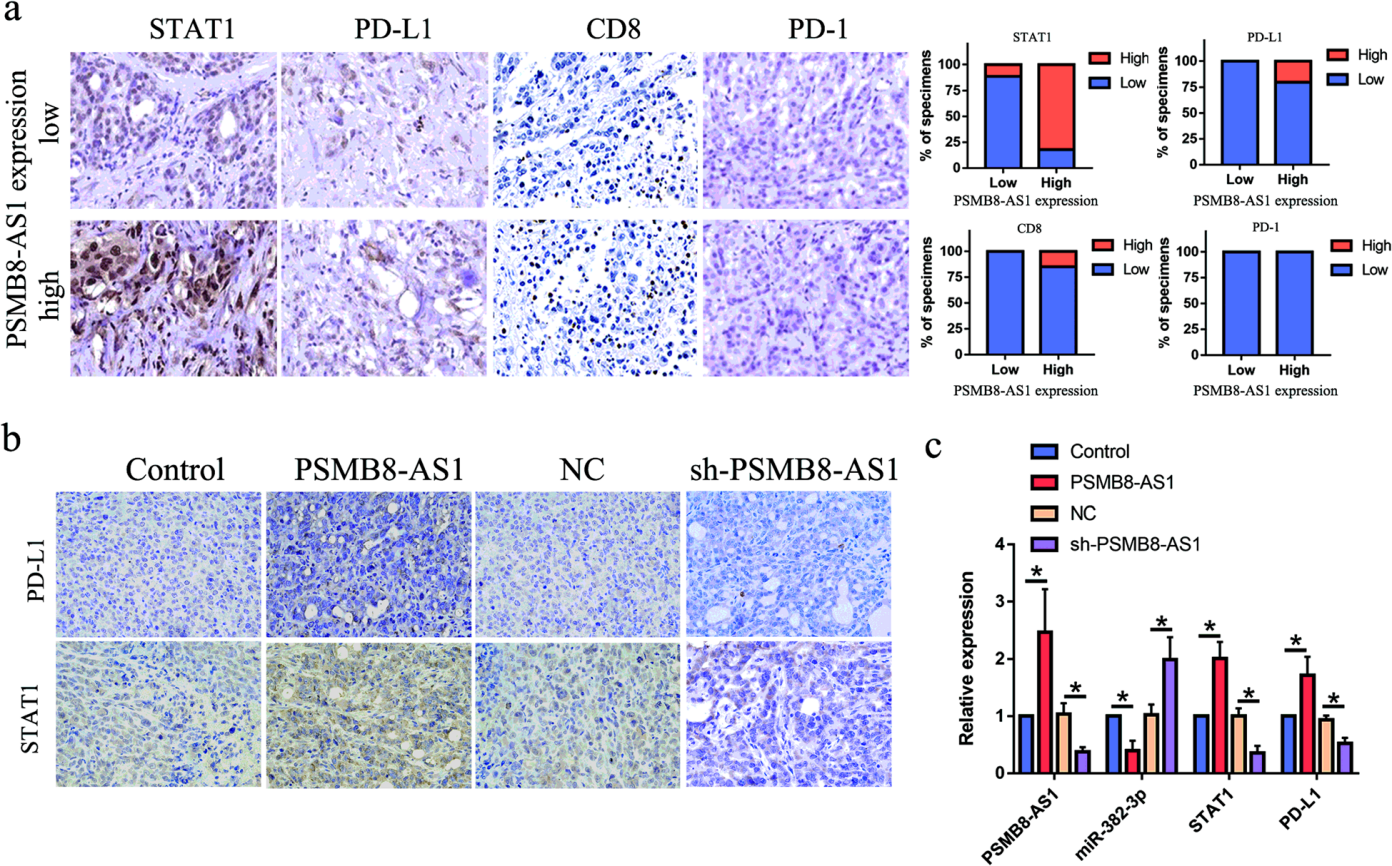
The positive correlation between PSMB8-AS1 and miR-382-3p/STAT1/PD-L1 axis in tumor tissues from xenografts and patients with PC. **a** IHC assay analysis the STAT1, PD-L1, CD8 and PD-1 in PC patients divided by PSMB8-AS1 expression. **b** IHC assay analysis the STAT1, PD-L1 expression in different group xenografts tissues. **c** RT-qPCR analysis of PSMB8-AS1, miR-382-3p, STAT1, PD-L1, expression in different group xenografts tissues

We already proved that PSMB8-AS1 can influence the miR-382-3p/STAT1/PD-L1 axis from the cell line. Then, we explored to verified the regulation in the PC and xenografts tissue. The IHC detection of the PC patient tissues demonstrated that STAT1、PD-L1and CD8 were significantly enhanced in the PSMB8-AS1 expressed higher specimens, and suppressed in the PSMB8-AS1 expressed lower specimens (Fig. 8a). As the expression of PD1 was almost all negative in the pancreatic cancer tissues, the expression of PD1 did not change significantly. The RT-qPCR was used to evaluate the expression level in different group xenografts tissue, the results indicated that PSMB8-AS1, STAT1, PD-L1 were upregulated in the PSMB8-AS1 overexpressed group, and downregulated in the PSMB8-AS1 knockdown group (Fig. 8b). IHC examination of the tumor xenografts eluted that the STAT1 and PD-L1 were enhanced in PSMB8-AS1 overexpressed group xenografts, whereas STAT1 and PD-L1 were markedly suppressed in the PSMB8-AS1 downregulated group (Fig. 8c). These data demonstrate that there is a significant positive correlation between PSMB8-AS1 and the miR-382-3p/STAT1/PD-L1 axis signaling pathway.

## Discussion

Accumulating evidence has demonstrated that lncRNAs play an important role in cancer progression. The molecular mechanisms underlying the lncRNA mediating malignant biological behavior and abnormal biological behavior remain unclear. In this study, we first analyzed the correlation between PSMB8-AS1 expression and pancreatic cancer clinical features. Further, we examined the role of PSMB8-AS1 in the progression of PC; function assays demonstrated that knockdown of PSMB8-AS1 significantly inhibited pancreatic cancer growth and metastasis. These results suggest that lncRNA PSMB8-AS1 exerts tumorigenesis in the progression of PC and could be a potential predictor of prognosis in PC patients. Giulietti M [14, 21] found that PSMB8-AS1 acts as an essential and potential prognostic biomarker and a therapeutic target using a systems biology approach on lncRNA expression data.

Furthermore, we explored the mechanism by which lncRNA PSMB8-AS1 promotes PC progression; we showed that PSMB8-AS1 acts as a miRNA sponge in PC to inhibit the function of miRNAs. As previously reported, lncRNAs could complete with the binding of miRNA to promote the target gene expression in transcription and translation [22–24]. Also, lncRNA/miRNA and the target gene-mediated ceRNA regulation network has been shown in PC progress [16, 18]. Our functional assay demonstrated that PSMB8-AS1 could act as the upstream gene of miRNA-382–3p. Furthermore, the luciferase reporter assay showed that miRNA-382–3p could directly combine with lncRNA PSMB8-AS1. Therefore, we speculated that lncRNA PSMB8-AS1 promoted PC progress by silencing the target genes of miR-382–3p. STAT1 is one of the target genes of miR-382–3p via the bioinformatic prediction. STAT1 is an important component in the NF-KB signal pathway, mediating cancer progression, and tumorigenesis [25, 26]. STAT1 usually acts as the transcriptional factor in the tumor immune function [27–29]. Studies demonstrated that STAT1 is the functional target of miRNAs [30–32] (miRNA-145–5p, miRNA-146A, miRNA-21), but the regulation of miRNA-382–3p and STAT1 remain unclear. To verify if STAT1 was the functional target of miR-382–3p, we first examined the expression and correlation of them. The result indicated that the expression of miR-382–3p was negatively correlated with STAT1, and significantly inhibited STAT1 expression in mRNA and protein level. Furthermore, the functional assay demonstrated that STAT1 could partly restrain the inhibition of miR-382–3p.

Accordingly, previous studies have revealed that PD-L1 could be transcriptionally activated by other transcriptional factors [33–35]. Previously, many experiments showed that STAT1 may transcriptionally activate PD-L1 in various types of cancers and mediated the inactivation of T cells [29, 36]. In addition, the oncogenic role of STAT1 on PC has been investigated by the previous studies as well [37–39]. These results mainly showed that STAT1 exerted an immune-associated function in cancer treatment and diagnosis. Afterward, we examined whether the PD-L1 expression was regulated by the ceRNA network. The results demonstrated that PD-L1 was transcriptional regulated by STAT1 and lncRNA PSMB8-AS1 in PC cells. Moreover, the functional assay also demonstrated that PD-L1 knockdown could restrain the oncogenic role of PC. In this study, we found that PD-L1 expression is positively correlated with lncRNA PSMB8-AS1, suggesting that PSMB8-AS1/miR-382–3p/STAT1 was an effective regulator of PC cells.

## Conclusion

This study demonstrates that PSMB8-AS1 improves the proliferation and metastasis of PC cells by sponging miR-382–3p to upregulate STAT1 expression. Ultimately, STAT1 transcriptionally activated PD-L1 and stability and exerted an immune associated function in pancreatic cancer, suggesting that PSMB8-AS1/miR-382–3p/STAT1/PD-L1 axis may act as a feasible therapeutic target for PC.

## Supplementary information

**Additional file 1: Table 1.** Association of PSMB8-AS1 expression with clinicopathological features of the PC patients.

**Additional file 2: Table 2.** The characteristics of the primers used in real-time PCR and plasmid sequence.

**Additional file 3: Fig. S1** a. Bioinformatic analysis of the correlation between PMSB8-AS1 and STAT1 in the TCGA database. b. Bioinformatic analysis of the correlation of STAT1 and PD-L1 in the TCGA database.

## Data Availability

The datasets used and/or analyzed during the current study are available from the corresponding author on reasonable request.

## Abbreviations

lncRNA: Long non-coding RNA
PC: Pancreatic cancer
miR: MicroRNA
qRT-PCR: Real-time quantitative reverse transcription PCR
CCK-8: Cell counting kit
