# Supplementary figures and images for: LncRNA PSMB8-AS1 contributes to pancreatic cancer progression via modulating miR-382-3p/STAT1/PD-L1 axis
Source: J Exp Clin Cancer Res. 2020 Sep 5;39:179. doi: 10.1186/s13046-020-01687-8 (PMC7487636; doi:10.1186/s13046-020-01687-8)

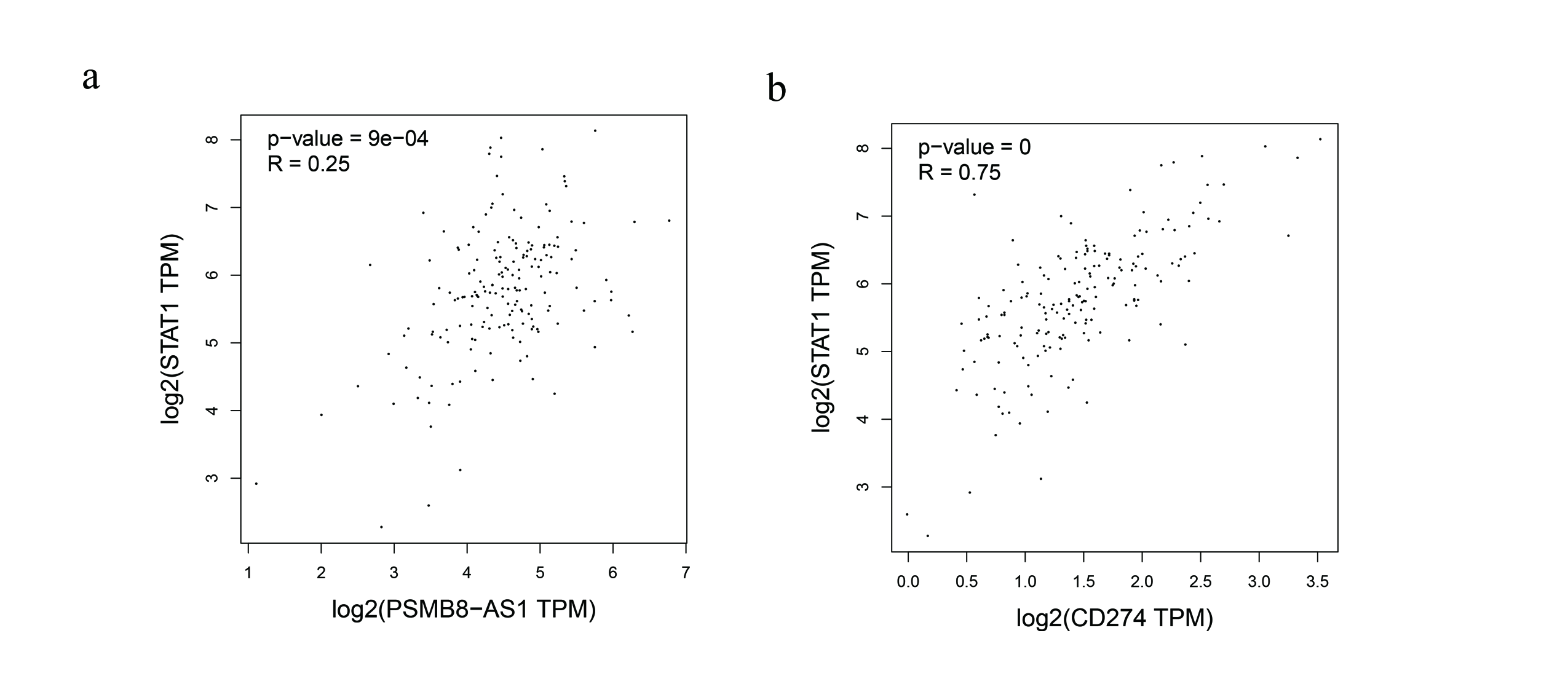

Supplement: Supplementary file 3 — Additional file 3: Fig. S1 a. Bioinformatic analysis of the correlation between PMSB8-AS1 and STAT1 in the TCGA database. b. Bioinformatic analysis of the correlation of STAT1 and PD-L1 in the TCGA database. [file 13046_2020_1687_MOESM3_ESM.tif]
